# Stroke care and outcomes in the Department of Neurology in Parakou, Benin: Retrospective cohort study

**DOI:** 10.1016/j.amsu.2020.07.041

**Published:** 2020-07-28

**Authors:** Thierry Adoukonou, Mendinatou Agbétou, Arlos Sowanou, Oyéné Kossi, Pervenche Fotso, Corine Houéhanou, Jean-Michel Vallat, Dismand Houinato, Pierre-Marie Preux, Philippe Lacroix

**Affiliations:** a Department of Neurology, University of Parakou, 03BP 10, Parakou, Benin; b Clinic of Neurology, University Teaching Hospital of Parakou, Benin; c U-1094 INSERM, University of Limoges, CHU Limoges, U-1094, Tropical Neuroepidemiology, Institute of Epidemiology and Tropical Neurology, GEIST, 87000, Limoges, France; d Department of Neurology, CHU Limoges Dupuytren, 87000, Limoges, France; e Department of Neurology, University of Abomey-Calavi, BP 188, Cotonou, Benin

**Keywords:** Stroke, Mortality, Benin

## Abstract

**Introduction:**

Stroke is one of the most common causes of high mortality rates in Africa with many unknown aspects around its prognosis. In this study we aim to describe stroke characteristics and in-hospital mortality of stroke in Parakou.

**Methods:**

This is a retrospective cohort study including all stroke patients admitted to the Department of Neurology at Parakou Teaching Hospital from January 1, 2013 through to December 31, 2019. Clinical data, vascular risk factors, stroke subtype and outcome data were recorded. The in-hospital case-fatality and its associated factors were determined. The study was approved by the Local Ethics Committee of Biomedical research and has been registered under the unique indentifying number researchregistry5687 and is available at https://www.researchregistry.com/browse-the-registry#home/

**Results:**

Stroke cases represented 51.5% of all patients. There were 372 patients included in the study with a mean age of 58.2 ± 14.2 years. The sex ratio was 1:3. Ischemic stroke accounted for 40.3%, intracerebral hemorrhage 30.4%, and unknown 29.3%. The main vascular risk factors were hypertension (69.1%), alcoholism (23.9%) and diabetes mellitus (16.9%). The mean NIHSS at admission was 9.4 ± 5.7 and the length of hospital stay was 9.0 ± 7.3. The most common complications recorded during the acute phase were swallowing disorders (10.2%), pneumonia (9.1%) and urinary tract infections (8.3%). The in-hospital case fatality was 6.2% and was associated with loss of consciousness (p = 0.0001), high NIHSS on admission (p = 0.001), fever (p = 0.0001), swallowing disorders (p = 0.001) and leukocytosis (p = 0.021). On discharge, 27.6% were independent and 97.8% were on antihypertensive drugs.

**Conclusion:**

The in-hospital stroke mortality was close to that reported by other studies in Africa.

## Highlights

• Subsaharan Africa (SSA) had a high burden of stroke.
• The stroke unit and stroke specialists can reduce the in-hospital mortality.
• We reported the low in-hospital mortality of stroke compared to those reported in SSA.
• The early detection and management of acute complications may reduce the acute complications of stroke.

## 1 Introduction

Stroke is a leading cause of death and has a high economic burden throughout the world [1]. Sub-Saharan African (SSA) countries have undergone an epidemiological transition and have a high burden of stroke and other non-communicable diseases. This geographical area is characterized by a lack of stroke specialists, stroke diagnostic facilities, and low availability of new stroke treatments [2,3]. Stroke was characterized by the high proportion of hemorrhage due to hypertension and small vessel disease in this geographical area [3]. Because of the scarcity of diagnostic tools and stroke specialists many stroke patients had unknown stroke subtypes in sub-Saharan Africa. Hypertension can explain more than 80% of all cases of stroke, and prevention needs to focus on stroke management. The management of the acute phase of stroke has not been compared in detail in those in western countries. The lack of facilities, lack of health insurance, misconceptions about stroke symptoms, cultural considerations and paucity of stroke specialists can explain the difference between high income countries and low and middle income countries [3]. Stroke mortality is also high in low income countries compared with those reported in western countries. Many studies have reported the in-hospital mortality of stroke in SSA with a range of 7.2%–45% [[4], [5], [6]]. We found an in-hospital case fatality of 16.8% in 85 cases [7]. Many factors have been reported to influence the outcome of stroke, particularly late admission, the acute complications of stroke and vascular risk factors. We aimed to describe the stroke pattern and in-hospital case fatality in a large cohort in Parakou University Hospital.

## 2 Methods

### 2.1 Registration and ethics

This section is written according to the STROCSS statement guidelines [8]. This study was approved by the Local Ethical Committee of Biomedical Research of the University of Parakou (Number 0300/CLERB-UP/P/SP/R/SA). Confidentiality of data was guaranteed in this study and it has been registered under the unique identifying number researchregistry5687 and is available at https://www.researchregistry.com/browse-the-registry#home/

### 2.2 Design

This was a single center retrospective cohort study.

### 2.3 Setting

The study took place in the Neurology Department of the University Hospital of Parakou. Many people in Benin do not have health insurance and do not present at the hospital. The city of Parakou is located 425 km north of Cotonou in Benin. The population is estimated at 255,478 inhabitants. The city has two large hospitals (a military hospital and a University teaching hospital). Those hospitals both received stroke patients but only the University hospital has neurologists and the beds dedicated for stroke patients. At the University hospital there are two neurologists, two cardiologists, one Intensive Care Unit (with three specialists), two neurosurgeons and a physiotherapy unit. The neurology department has two neurologists and 10 beds with two dedicated to stroke. The two dedicated stroke beds are scoped beds with 24-h multi-parameter monitoring (blood pressure, oxygen saturation, pulsation, heart rhythm, temperature) with access to oxygen and a mucus aspirator, and the beds have anti-decubitus mattresses. The room with two beds is intended for subjects in the acute phase of stroke. However, patients past that phase, as well as those admitted after 3 days and who are stable, are hospitalized in the other beds of the service.

Protocols drawn up in accordance with international recommendations are available and accessible in the service. There is a 24-h nurse and two caregivers for all 10 beds. The medical staff is made up of two neurologists, including a professor specializing in stroke, two general practionners and two physiotherapists. All service personnel were trained beforehand regarding stroke management.

Modern treatments such as thrombolysis and thrombectomy are not available, and despite the availability of rt-PA in the hospital for the prior six months, no intravenous thrombolysis had been performed. Osmotic drugs, intravenous antihypertensive drugs, low weight molecular heparin, acetaminophen, aspirin, clopidogrel, and statins are some of the drugs that are available. Few people in Benin have health insurance, which means that many patients cannot benefit from additional diagnostic or etiological examinations.

### 2.4 Cohort group

All stroke patients admitted between January 1, 2013 to December 31, 2019 were included and followed-up until hospital discharge.

## 3 Participants

Inclusion criteria: All stroke cases met the inclusion criteria. The diagnosis of stroke was based on the World Health Organization (WHO) criteria [9] or on brain CT-scan results. Stroke was defined as “rapidly developing signs of focal (or global) disturbance of cerebral function, leading to death or lasting longer than 24 h, with no apparent cause other than vascular” [9]. The patients with transient ischemic attack, those who died prior to evaluation and others with subarachnoid hemorrhage were excluded.

### 3.1 Sampling

All stroke cases were included and were recruited systematically

### 3.2 Intervention

Sociodemographic data, vascular risk factors, and clinical data were recorded upon admission. The vascular risk factors were defined according to WHO stepwise approach [10].

The initial neurologic impairment assessment was based on the National Institute of Health Stroke Scale (NIHSS). We recorded the systolic and diastolic blood pressure and temperature. Some patients had laboratory tests (blood sugar, acid uric, blood cell count, cholesterol, triglycerides) and cardiac explorations (electrocardiogram or echocardiography) or ultrasound of neck vessels for the etiologic investigation. We systematically recorded the stroke subtype as ischemic, hemorrhagic, and unknown (without CT scan); the medical complications that occurred during acute phases were pneumonia, swallowing disorders, urinary tract infection, and seizure. The diagnosis of each complication was made by a neurologist based on the clinical presentation and the results of biological tests and other examinations.

The swallowing test was systematically carried out by the doctor upon admission to the service. A nasogastric tube was placed and monitored daily for those with swallowing disorders.

Diagnostic examinations (CT scan, cardiac explorations, biological tests, etc.) are routinely prescribed to all patients depending on the type of stroke. However, in the absence of insurance and medical coverage, only patients with the financial resources can carry them out.

Management was performed according to the international recommendations regarding the local protocol for each stroke subtype.

The patients with good improvement following an acute phase were released to home but those who worsened were sent to the intensive care unit. Our physiotherapy department cannot admit patients for hospitalization; the main issues were death, transfer to another unit or home discharge.

### 3.3 Outcome

We recorded the outcome of each patient and the length of hospital stay. The primary outcome was the stroke case fatality on discharge.

### 3.4 Statistical methods

The data were analyzed using Statistical Package for Social Sciences (SPSS) version 21.0 software (IBM) and the usual parameters were used to describe the variables. We compared the different types of stroke according to other variables and the associated variables for in-hospital death. We used the chi-2 test (or Fisher test) for the percentages and the Student t-test to compare the quantitative variables. A p value of 0.05 was considered to be significant.

## 4 Results

**Fig. 1:**
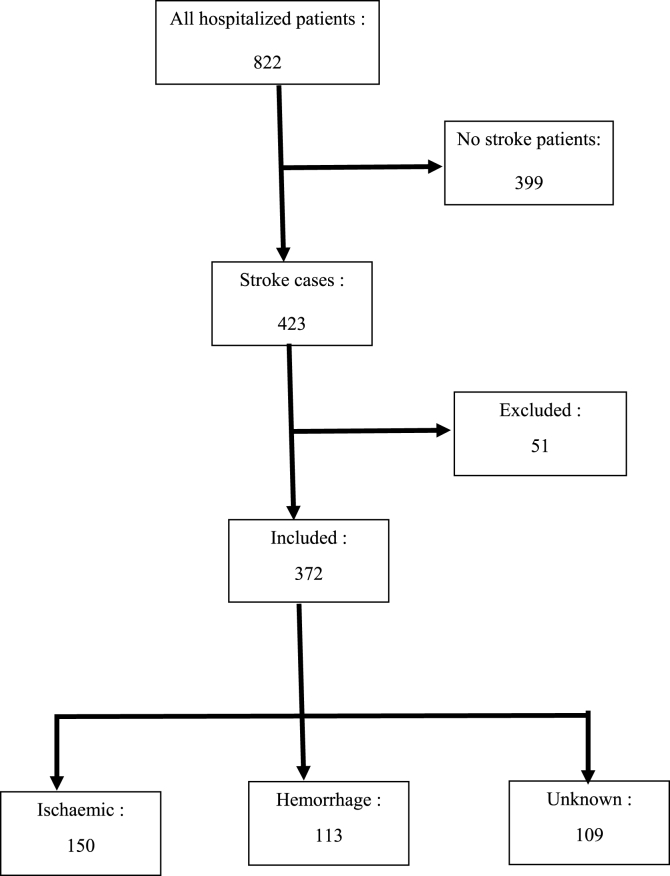
Flow chart of the patients selection.

Four hundred twenty-three stroke cases were recorded with an incidence of 51.5% (423/822) of all hospitalizations during the study period. Among the 423 strokes cases, 372 were included in this study (51 patient records were unusable or lost of their medical sheet) with a participation rate of 87.9% (cf. Fig. 1). The patients were 16–97 years of age with a mean of 58.2 ± 14.2 years. The sex ratio was 1:3 (210 were male). The median time to admission since the onset of symptoms was 48 h with an interquartile interval of [20–96 h]. Only 10.3% of patients were admitted to hospital within 3 h. The characteristics of the patients are summarized in Table 1 according to vascular risk factors and clinical and biological data. The greatest vascular risk factor was hypertension (69.1%) followed by alcoholism (23.9%) and diabetes mellitus (16.9%). There was no difference in the frequency of vascular risk factors in the three stroke types. Ischemic stroke cases accounted for 40.3%, hemorrhage for 30.4% and unknown subtype for 29.3%. The patients with ischemic stroke were older and had lower admission blood pressure than others. In fact, the ischemic stroke patients had a mean blood pressure of 151.3 ± 31.7 mm Hg and 94.0 ± 19.4 mm Hg, respectively, for systolic and diastolic. Those values were significantly lower than those in hemorrhagic and unknown stroke subtype (p = 0.002 for systolic and 0.0001 for diastolic). Admission blood glucose was higher in patients with unknown stroke, although the proportion of diabetics was the same in all three groups (Table 1). The patients with intracerebral hemorrhage stayed in hospital longer than the other patients.

**Table 1:** Main characteristics of stroke patients, Parakou, 2020.

	All patients (N = 372)	Ischaemic stroke (N = 150)	ICH (N = 113)	Unknown stroke (N = 109)	p
**Sex** (%male)	210 (56.5)	84 (56.0)	70 (61.9)	56 (51.4)	0.28
Female	162 (43.5)	66 (44.0)	43 (38.1)	53 (48.6)	
**Age** (mean± standard deviation)	58.2 ± 14.2	61.7 ± 14.2	52.6 ± 10.9	59.2 ± 15.4	**0.0001**
**Vascular risk factor n (%)**					
Hypertension	257 (69.1)	103 (68.7)	77 (68.1)	77 (70.6)	0.912
Alcoholism	89 (23.9)	31 (20.7)	29 (25.7)	29 (26.6)	0.474
Diabetes mellitus	63 (16.9)	29 (19.3)	14 (12.4)	20 (18.3)	0.297
Smoking	52 (14.0)	20 (13.3)	13 (11.5)	19 (17.4)	0.426
Previous stroke	53 (14.3)	24 (16.2)	10 (8.8)	19 (17.4)	0.132
Dyslipidemia	14 (3.8)	9 (6.0)	3 (2.7)	2 (1.8)	0.167
Transient Ischemic attack	6 (1.6)	2 (1.3)	3 (2.7)	1 (0.9)	0.555
Heart disease	16 (4.3)	5 (3.5)	4 (3.5)	7 (6.4)	0.429
Migraine	44 (11.8)	19 (12.7)	18 (15.9)	7 (6.4)	0.083
**Complications during hospitalization**					
Pneumonia	34 (9.1)	16 (10.7)	10 (8.8)	8 (7.3)	0.651
Swallowing disorders,	38 (10.2)	19 (12.7)	12 (10.6)	7 (6.4)	0.258
Seizures	20 (5.4)	6 (4.0)	8 (7.1)	6 (5.5)	0.547
Urinary tract infection	31 (8.3)	18 (12.0)	9 (8.0)	4 (3.7)	0.056
**Clinical data at admission**					
Glasgow (mean ± SD)	13.2 ± 2.5	13.5 ± 2.1	13.2 ± 2.5	12.9 ± 3.0	0.241
NIHSS at admission (mean ± SD)	9.4 ± 5.7	9.6 ± 5.8	9.9 ± 5.6	8.4 ± 5.8	0.313
Systolic BP (mean ± SD)	157.6 ± 33.0	151.3 ± 31.7	166.1 ± 31.7	157.3 ± 34.5	**0.002**
Diastolic BP (mean ± SD)	98.7 ± 20.3	94.0 ± 19.4	104.1 ± 16.8	99.4 ± 23.3	**0.0001**
Temperature ont admission (mean ± SD)	37.0 ± 0.6	37.0 ± 0.6	37.1 ± 0.7	37.0 ± 0.7	0.568
Glycemia (mean ± SD)	1.3 ± 0.6	1.2 ± 0.7	1.1 ± 0.3	1.4 ± 0.8	**0.007**
**Biological data (Mean ± SD)**					
Haemoglobin rate (243) (g/dl)	13.6 ± 7.9	14.6 ± 11.3	13.4 ± 3.6	11.7 ± 2.3	0.095
Platelet count (214) 10^3^ cells	164.5 ± 135.5	160.2 ± 143.1	161.5 ± 134.7	178.9 ± 121.7	0.730
Total Cholesterol (213) (g/l)	1.8 ± 0.5	1.7 ± 0.5	1.8 ± 0.5	1.8 ± 0.5	0.739
Triglycerid (201) (g/l)	1.0 ± 0.5	1.0 ± 0.5	0.9 ± 0.5	1.1 ± 0.5	0.309
Uric acid (44)	58.1 ± 30.4	63.9 ± 36.0	51.7 ± 23.6	51.5 ± 15.9	0.420

The NIHSS ranged from 0 to 29 with a mean of 9.4 ± 5.7. In patients with ischemic stroke the locations were middle cerebral artery (67.4%); anterior cerebral artery (14.2%), posterior cerebral artery (4.2%), vertebro-basilar territory (5.0%) and multifocal sites (9.2%). However, deep location (77.5%) followed by lobar location (19.1%) and brainstem (3.4%) was the most frequent site of intracerebral hemorrhage. Many acute complications were recorded during hospitalization: 9.1% had pneumoniae, 10.2% swallowing disorders, 20 (5.4%) patients had seizures and 31 patients (8.3%) had urinary tract infection.

Among the patients only 121 (32.5%) had EKG; 31 (8.3%) had cardiac echography and 34 had ultrasound echography of neck vessels (9.1%). Four cases of atrial fibrillation were detected from the EKG data of ischemic stroke patients. The outcomes are listed in Table 2. Upon discharge, patients with ischemic stroke were taking more antiplatelet drugs and statins than the other patients, but only 132 (88.0%) of them had antiplatelet. Four patients had anticoagulant on discharge after antiplatelet in the acute phase. The use of an antiplatelet drug differed for others due to the hemorrhagic transformation of the infarction in the acute phase. Surprisingly, one patient with hemorrhagic stroke used one 75 mg aspirin per day on discharge due to the cardiac prescription (he accumulated four vascular risk factors such as hypertension, diabetes, previous myocardial infarction and a minor hemorrhage on brain CT scan). The antihypertensive drugs were prescribed more to patients with intracerebral hemorrhage (ICH). Twenty-three deaths were recorded with an in-hospital mortality of 6.2% during the hospitalization period. Disorders of consciousness, high neurological impairment, fever on admission, swallowing disorders and hyperleucocytosis were the associated factors for death (Table 3). Upon discharge, among the 349 survivors, 27.6% were independent (Rankin ≤2), 23 (6.5%) had RANKIN 0, 40 (11.4%) had RANKIN 1; 54 (15.4%) had RANKIN 3, 133 (38.2%) had RANKIN 4 and 62 (18.7%) had 5. The median RANKIN was 4 and the interquartile interval was [[2], [3], [4]].

**Table 2:** Discharge characteristics of stroke patients, Parakou 2020.

	All patients (N = 372)	Ischaemic stroke (N = 150)	ICH (N = 113)	Unknown stroke (N = 109)	p
**Outcome data**					
Length of hospital stay (days)	9.0 ± 7.3	9.6 ± 6.9	11.8 ± 8.1	5.4 ± 5.4	**0.0001**
In-hospital death	23 (6.2)	11 (7.3)	7 (6.2)	5 (4.6)	0.663
**Treatment at hospital Discharge (%)**					
Antihypertensive drugs	327 (87.9)	127 (84.7)	109 (96.5)	90 (82.6)	0.074
Antiplatelet	163 (43.8)	132 (88.0)	1 (0.9)	30 (27.5)	**0.0001**
Antidiabetic drugs	36 (9.7)	15 (10.0)	7 (6.2)	15 (13.8)	0.424
Statins	50 (13.5)	36 (24.0)	9 (7.9)	16 (14.7)	**0.015**
Physiotherapy	228 (61.3)	103 (68.7)	85 (75.2)	40 (36.7)	**0.0001**

**Table 3:** In-hospital mortality of stroke patients, Parakou 2020.

	Alive (N = 349)	Dead (N = 23)	p
**Sexe** (Male%)	195 (55.9)	15 (65.2)	0.257
**Age** (years) Mean ± SD	58.3 ± 14.2	57.6 ± 14.4	0.830
**Vascular risk factor n (%)**			
Hypertension	7 (69.1)	16 (69.6)	0.582
Alcoholism	82 (23.5)	7 (30.7)	0.298
Diabetes mellitus	58 (16.6)	5 (21.7)	0.346
Smoking	50 (14.3)	2 (8.7)	0.350
Previous stroke	50 (41.4)	3 (13.0)	0.576
Dyslipidemia	14 (4.0)	0 (0.0)	0.403
Transient ischemic attack	6 (1.7)	0 (0.0)	0.680
Heart disease	16 (4.6)	0 (0.0)	0.352
Migraine	42 (12.0)	2 (8.7)	0.473
**Complications during hospitalization**			
Pneumonia	30 (8.6)	4 (17.4)	0.147
Swallowing disorders,	30 (8.6)	8 (34.8)	**0.001**
Seizures	19 (5.4)	1 (4.3)	0.650
Urinary tract infection	27 (7.7)	4 (17.4)	0.113
**Clinical data on admission**			
Glasgow (mean ± SD)	13.4 ± 2.4	10.6 ± 3.4	**0.0001**
NIHSS at admission (mean ± SD)	9.1 ± 5.6	13.7 ± 7.1	**0.005**
Systolic BP (mean ± SD)	158.5 ± 32.9	144.6 ± 32.6	0.051
Diastolic BP (mean ± SD)	99.2 ± 20.2	92.1 ± 21.2	0.104
Temperature on admission (mean ± SD)	37.0 ± 0.6	37.5 ± 0.7	**0.0001**
Glycaemia (mean ± SD)	1.3 ± 0.7	1.3 ± 0.4	0.743
Leucocyts (mean ± SD) 10^3^ cells/mm^3^	10.6 ± 12.4	18.1 ± 19.3	**0.021**
**Length of hospital stay**	9.2 ± 7.4	6.2 ± 6.4	0.057

### 4.1 Key results

Hypertension was the greatest risk factor and ischemic stroke accounted for 40.3% of all strokes. Mean patient age was 58.2 ± 14.2 years. The in-hospital mortality in this setting was 6.2% and was significantly associated with the high neurological impairment, fever on admission, swallowing disorders and hyperleucocytosis.

## 5 Discussion

We aimed to describe stroke characteristics and in-hospital mortality at the University Hospital of Parakou over a 6-year period (2013–2019).

Stroke was the leading cause of hospitalization in neurology and accounted for half (51.5%) of all hospitalizations. This finding is consistent with all studies on the increasing prevalence and incidence of stroke worldwide, especially in sub-Saharan Africa [11]. Nevertheless, the exclusion of 51 patients can induce bias, although in the registry of the death, no patients died during hospitalization. We can postulate that if we take them into account the case fatality would be lower than what we reported. On the other hand, many patients did not present to the hospital and the data we reported here cannot be generalized to all patients in the city. We think that it is very important to build an urban registry of stroke in this city to appreciate the real stroke mortality.

Contrary to European literature [12], in our study, ICH is more common (ischemic stroke 40.32% versus ICH 30.37%) in SSA. Mapouré et al. reported 52% ischemic stroke and 48% ICH. This is probably linked to the early incidence and severity of arterial hypertension in the black African population, responsible for lipohyalinosis of the small arteries with fibrinoid necrosis, which in turn promotes the formation of micro-aneurysms which occasionally break up under high blood pressure [13]. On other hand, most of the patients with hypertension were unaware of their status before stroke onset, and may be why the patients with ICH had higher blood pressure on admission than did the other patients. The management of high blood pressure often requires longer time frames to obtain optimal values and can explain the length of hospital stay of the patients with ICH. Those patients were prescribed additional antihypertensive drugs on discharge. Hypertension is the most common risk factor (69.1%) irrespective of the type of stroke (68.7% ischemia versus 68.1% hemorrhage), followed by alcoholism and diabetes. This observation is comparable to the results of other African authors. Indeed, these countries have vascular similarities with our observations.

The overall mortality was 6.2%. Mortality rates are rather disparate in the various African and Western publications. Mapouré et al. in Douala reported in-hospital mortality of 26.8%, Touré et al. reported 24.84% in Senegal in 2010 [14,15]. Other sub-Saharan African authors have described a mortality of 9.5 and 15.5% in patients followed-up exclusively in a Neurology clinic, as in our study [16,17]. Western studies that included both ischemic and hemorrhagic strokes reported in-hospital mortality rates of between 4.8% [18] and 16% [19]. A multicentric Asian study similar to the current study reported 12.5% [20]. These differences could be explained by the quality of the global healthcare system: systematic screening of cardiovascular conditions, the delays in management, and mostly the universal access to healthcare [13].

Accessibility to the tools of exploration for stroke remains a real problem with wide social inequalities and health insurance benefiting mostly the wealthy. Among the patients: 109 (29.3%) unknown stroke, only 121 (32.5%) had an EKG; 31 (8.3%) benefited from cardiac echography and 34 ultrasound echography of neck vessels (9.1%). This undoubtedly affects the prognosis of the patients who do not receive optimal and specific treatment. In secondary prevention only 23.7% of ischemic stroke were taking statins. In our current practice the use of statins depends on the level of LDL-cholesterol. In fact, only patients with LDL-cholesterol higher than 1 g/l require statins. The low rate of patients on statins can be explained by the lack of biological tests (no insurance).

Swallowing disorders, pneumonia and urinary tract infections were the most common complications in the deceased. Similar results have been reported by Mapouré and Langhorne et al. [14,19]. Inhalation pneumonia complicated swallowing disorders and is favored by the feeding of patients by their families often without authorization from the nursing staff. Urinary tract infections often complicated prolonged decubitus and co-morbidities such as diabetes. Touré et al. also reported that long hospital stays were a predictor of mortality [15]. The hyperleucocytosis was also associated to the in-hospital mortality. Patients with pneumonia and urinary tract infections had infection and the usual biological marker of infectious diseases is hyperleucocytosis.

The existence of consciousness disorders was a predictor of mortality in our study. Touré et al., and Mapouré et al. made the same observation [14,15]. Consciousness disorders not only induce cardio-respiratory distress, but also promote pneumonia. Other poor predictors of mortality were poor neurological status assessed by the NIHSS, fever on admission, and leukocytosis probably stemming from infectious complications which shows the importance of actively seeking out infectious complications, and preventing and treating them early to avoid deleterious development.

### 5.1 Strengths and limitations

The low rate of in-hospital mortality was probably due to availability of a stroke specialist (TA) and the systematic detection of swallowing disorders and other acute stroke complications. However, the low percentage of patients benefiting from CT scan and the low rate of etiological explorations may present limitations to our findings.

## 6 Conclusion

The in-hospital mortality of stroke was comparable to other reports in African countries. It could be explained by acute complications such as pneumonia due to swallowing disorders. The main strategy to reduce the in-hospital stroke mortality is the detection and the treatment of the acute complications.

## Funding

No funding.

## Ethical approval

This study was approved by the Local ethical committee of Biomedical research of University of Parakou Number 0300/CLERB-UP/P/SP/R/SA.

## Author contribution

All authors contributed equally in the writing of this manuscript.

## Registration of research studies

This study has been registered under the unique indentifying number researchregistry5687 and is available at https://www.researchregistry.com/browse-the-registry#home/

## Guarantor

Thierry ADOUKONOU.

## Provenance and peer review

Not commissioned, externally peer reviewed.

## Declaration of competing interest

No conflict of interest.
